# Aging, Neurodegenerative Disorders, and Cerebellum

**DOI:** 10.3390/ijms25021018

**Published:** 2024-01-13

**Authors:** Igor Y. Iskusnykh, Anastasia A. Zakharova, Evgenii D. Kryl’skii, Tatyana N. Popova

**Affiliations:** 1 Department of Anatomy and Neurobiology, University of Tennessee Health Science Center, Memphis, TN 38163, USA; 2 Department of Medical Biochemistry, Faculty of Biomedicine, Pirogov Russian National Research Medical University, Ostrovitianov St. 1, Moscow 117997, Russia; 3 Department of Medical Biochemistry, Molecular and Cell Biology, Voronezh State University, Universitetskaya Sq. 1, Voronezh 394018, Russia; krylskiy@bio.vsu.ru (E.D.K.);

**Keywords:** cerebellum, neurodegeneration, aging, brain, neuron, Purkinje cells, granule cells, inflammation, Alzheimer’s disease, dementia

## Abstract

An important part of the central nervous system (CNS), the cerebellum is involved in motor control, learning, reflex adaptation, and cognition. Diminished cerebellar function results in the motor and cognitive impairment observed in patients with neurodegenerative disorders such as Alzheimer’s disease (AD), vascular dementia (VD), Parkinson’s disease (PD), Huntington’s disease (HD), spinal muscular atrophy (SMA), amyotrophic lateral sclerosis (ALS), Friedreich’s ataxia (FRDA), and multiple sclerosis (MS), and even during the normal aging process. In most neurodegenerative disorders, impairment mainly occurs as a result of morphological changes over time, although during the early stages of some disorders such as AD, the cerebellum also serves a compensatory function. Biological aging is accompanied by changes in cerebellar circuits, which are predominantly involved in motor control. Despite decades of research, the functional contributions of the cerebellum and the underlying molecular mechanisms in aging and neurodegenerative disorders remain largely unknown. Therefore, this review will highlight the molecular and cellular events in the cerebellum that are disrupted during the process of aging and the development of neurodegenerative disorders. We believe that deeper insights into the pathophysiological mechanisms of the cerebellum during aging and the development of neurodegenerative disorders will be essential for the design of new effective strategies for neuroprotection and the alleviation of some neurodegenerative disorders.

## 1. Introduction

The incredibly intricate human central nervous system (CNS) consists of distinct cell types that form hundreds of functional and anatomical structures of the brain and spinal cord. Comparative studies of brain evolution mostly focus on its unique anatomical structures, the best example of which is the significant evolutionary expansion of the cerebral cortex that leads to the heightened cognitive function in humans. Unfortunately, the mammalian structures that are the most anatomically conserved are studied less because they are perceived to be functionally too similar between mammal species. One such structure is the cerebellum, which has recently been associated with a wide range of functions that are impaired during aging and in neurodegenerative disorders [1]. The human cerebellum is involved in a wide range of functions, including motor control, perception, language, working memory, cognitive control, and social cognition. The literature suggests that the cerebellum exhibits functional heterogeneity, both in terms of task-induced activity patterns and functional connectivity with the neocortex measured in the absence of a task. The cerebellum may contribute to the formation of cortical circuits during certain sensitive stages of human development in the cognitive and social domains [2,3]. Although numerous disorders ranging from ataxia to neurodegenerative disorders are associated with the human cerebellum [4], the cellular and developmental processes that give rise to the cerebellum have not been studied sufficiently. Thus, the role of the human cerebellum in aging and neurodegenerative disorders remains poorly understood despite a long history of studies on model vertebrates.

**Figure 1:**
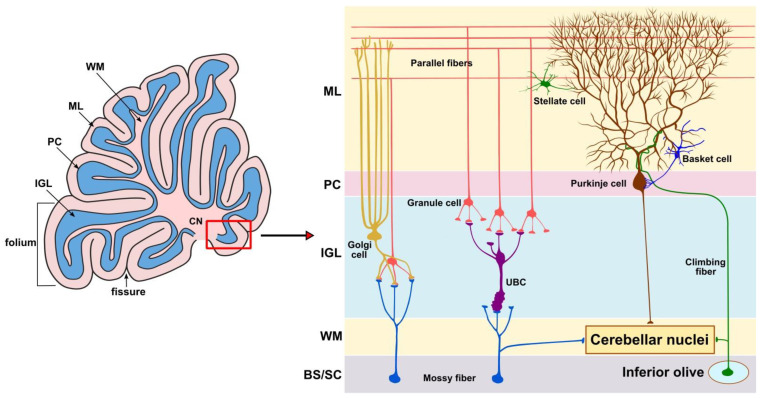
**Schematic representation of a sagittal section of the cerebellum showing its organization, including cerebellar cells and interconnections between fibers.** In the **left panel**, the fissure is indicated by an arrow, and the folium by a straight bracket. The ML is indicated by the outermost pink band, the PC by the black line, the IGL by the blue band, and the WM by the inner pink layer. In the **right panel**, a higher magnification of the area boxed in red shows the organization of the layers. Abbreviations used: BS/SC, brainstem/spinal cord; CN, cerebellar nuclei; ML, molecular layer; PC, Purkinje cell layer; IGL, internal granule layer; UBC, unipolar brush cells; WM, white matter.

The morphology of the cerebellar cortex is organized similarly across mammal species. Anatomically, it is divided into two lateral hemispheres and a vermis that is located in the cerebellar midsagittal plane between the hemispheres. As shown schematically in Figure 1, the cerebellum in adults comprises three layers: the molecular layer (ML), which includes basket and stellate cells; the Purkinje cell layer, which contains Purkinje cells (PC); and the granule cell layer, which includes granule cells (GCs), unipolar brush cells, Lugaro cells, and Golgi cells [5,6,7,8,9,10,11,12]. The immature cerebellum also includes the outermost external granule layer (EGL) that is formed by small, densely packed cells. The temporary EGL produces the GCs that comprise the largest population of neurons [13,14].

There is now an increasing acknowledgment that the functional impairment of cognitive and motor functions in aging might be related to the neurodevelopmental processes by which the cerebellum arises [15,16]. It is clear that proper neurodevelopment is essential for the formation of an adequate cell reserve with appropriate cellular diversity to limit the adverse effects of neuronal loss during aging [17,18]. However, our understanding of how exactly early developmental factors may impact the cerebellum and cognition throughout the human lifespan remains incomplete.

**Figure 2:**
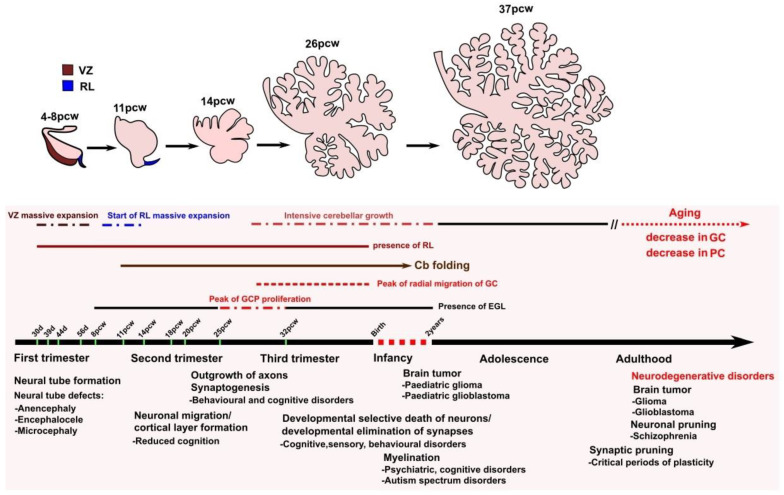
**A timeline of cerebellar development and diseases related to brain development across the human lifespan.** Shown are the events of cerebellar development and examples of the related diseases that occur at different perinatal and postnatal stages of human development. Abbreviations used: Cb, cerebellum; GC, granule cells; GCP, granule cell progenitors; PC, Purkinje cells; PCW, post-conception weeks; RL, rhombic lip; VZ, ventricular zone.

The cerebellar neurons are derived according to precise timetables of neurogenesis. The timeline in which this occurs in humans is shown in Figure 2 [19,20,21]. During embryonic development, the cerebellum arises from rhombomere 1, the most anterior segment of the hindbrain [22,23]. The isthmic organizer located at the boundary between the mesencephalon and rhombomere 1 directs the formation of the cerebellar territory [24]. Within the cerebellar territory are established two germinal zones with distinct development potentialities, the ventricular neuroepithelium (ventricular zone) and the anterior rhombic lip, from which all the GABAergic neurons, such as PCs, the nucleo-olivary projection neurons of the deep cerebellar nuclei (DCN), and the inhibitory interneurons, originate from the ventricular zone [25], while the glutamatergic neurons, including most projection neurons of the DCN and unipolar brush cells (UBCs), arise from progenitors in the anterior rhombic lip [26,27]. The precursors of GCs also emerge from the rhombic lip but migrate tangentially over the pial surface to form the secondary matrix called the EGL [13], where they proliferate under the influence of sonic hedgehog (Shh) and Jag1 proteins that are produced by Purkinje cells. The GC precursors then migrate radially along Bergmann glial fibers to form the internal granule cell layer (IGL) below the Purkinje cell layer. In humans, maximal granule cell proliferation, migration of differentiated granule neurons to the IGL, and maturation of Purkinje cells occur during the third trimester of pregnancy [5,28,29].

In recent years, increasingly greater attention has been paid to the role of the cerebellum in aging and neurodegenerative disorders [16,30,31], while the number of clinical studies that describe the incidence of brain disorders associated with the aging cerebellum has dramatically increased [32,33,34,35]. Thus, investigating new mechanisms involved in aging and the development of neurodegenerative disorders in the cerebellum is important from both a fundamental and clinical standpoint. To promote a better understanding of the timing and patterns of aging-related issues that affect the cerebellum, we emphasize the need to characterize the aging process in different neurodevelopmental disorders.

## 2. The Role of the Cerebellum in Aging

Changes in motor coordination and cognitive function associated with cerebellar neuronal dysfunction are among the most important indicators of aging [36,37,38]. The cerebellum of aged mice contains a varying number and length of junctional breaks that result in partial or total loss of blood–brain barrier (BBB) [39], and the macrostructure of cerebellar neurons changes with age. A significant age-related decline in the cerebellar vermal region (lobule VIIA) of healthy human volunteers was detected by analyzing the intra-neurite volume fraction and neurite orientation-dispersion index demonstrated, which might be related to synaptic and dendritic deficits. These differences might be mediated by the compensatory growth of a dendritic tree in this cerebellar region in response to the partial deafferentation of other brain areas [40]. Aging is also accompanied by the loss of cerebellar cells through cellular necrosis and iron-dependent ferroptosis [41,42]. An intensification of oxidative processes causes DNA damage, and the variance of DNA modifications is increased in the cerebellum of aged volunteers, measured statistically as age-dependent heteroscedasticity [43]. 

Aging is associated with progressive mitochondrial dysfunctionin the cerebellum. During aging an impaired function of small and medium-sized mitochondria results in significant decay of energy metabolism. [44]. Numerous proteins in mitochondria are expressed ubiquitously and control mitochondrial fusion/fission dynamics. Their dysfunction is associated with several diseases. Age-related disorders, such as postural control dysfunction, cerebellar ataxia, essential tremor, Alzheimer’s disease, and Lewy body disease, may be responsible for the mechanisms of age-related cerebellar PC degeneration [45]. Zhang et al. modeled the loss of GABA α1-receptor subunits and the upregulation of α2/3-receptor subunits in the dentate nucleus of the cerebellum. They found that changes in the amplitude and decay time of GABAergic currents in the dentate nucleus could contribute to sustained oscillatory activity at the essential tremor frequency [46].

Evidence suggests that the activity of complex I decreases in the cerebellum of mice with age. This decrease is significantly greater in mice with simulated cerebellar ataxia with neurodegeneration [45]. Popa-Wagner et al. discovered a significant increase in the turnover rate of several electron-transport chain proteins, particularly complex III (ubiquinol-cytochrome C oxidoreductase) and complex IV (cytochrome C oxidoreductase), in the cerebellum of aged mice. At the same time, the turnover rate of proteins involved in mitophagy did not significantly change with age [45]. Age-related changes in calcium homeostasis are a characteristic of cerebellar neurons. This is manifested by a delayed recovery of intracellular calcium concentration after intensive stimulation. The delay is caused by significant changes in the metabolic status of mitochondria, which lead to a lag in the depolarization–repolarization cycle of the mitochondrial membrane potential. The rate of mitochondrial repolarization decreases in old neurons, which may result in a reduced ability of mitochondria to produce enough ATP to meet energy demands [47]. These data are consistent with the findings of Turpeenoja et al. who demonstrated several statistically significant quantitative changes in the proteins of the inner mitochondrial membrane in the cerebellum of aged rats. Specifically, they observed a decrease in the number of proteins with molecular masses of 75 kDa, 16 kDa, and 14 kDa, corresponding to NADH-dehydrogenase or cytochrome a (75 kDa), cytochrome oxidase subunit IV (16 kDa), and cytochrome c (14 kDa). The reduction in the levels of the aforementioned proteins may be linked to a decline in the transcriptional activity of the nuclear genome or to a disruption of the structure of the mitochondrial membrane, which could involve alterations in membrane permeability or an increase in membrane rigidity during the aging process [48]. Balietti et al. demonstrated a gradual reduction in the numerical density of mitochondria that are positive for succinate dehydrogenase during cerebellar aging [49]. However, it should be noted that while the levels of proteins involved in energy metabolism pathways do change significantly with age, the cerebellum has a stable mitochondrial proteome compared to the cortex and hippocampus [50].

The gene expression profile in the cerebellum is also altered by aging. In the cerebellum of aged female rats, the activity and protein level of the cytochrome P450 family member CYP2D were decreased, possibly indicating a lower level of brain metabolism [51]. During the aging process, the normally high levels of intracellular calcium channels such as the ryanodine receptors (RyRs) that are expressed in Purkinje cells and the granular layer drop significantly [52]. Such reduction in the receptor expression might adversely affect cerebellar Ca^2+^ signaling and synaptic function [53]. Interestingly, the cerebellum of patients with AD demonstrated no visible pathological changes, only moderate alterations in the levels of synaptic proteins, few amyloid plaques, and a relatively equal amount of the Aβ isoforms Aβ40 and Aβ42 [54]. Similarly, aged female Lewis rats demonstrated no age-dependent changes in citrate synthase (CS) activity or the protein levels or enzymatic activity of mitochondrial complexes I–V, all of which serve as markers of mitochondrial biogenesis, mitophagy, fusion, and fission of cerebellum [55]. The multidirectional cerebellar changes described in the literature might be related to the cell-specificity of age-induced modifications. For example, glial cells in the cerebellar ML become smaller yet more numerous with age [56]. 

In aging healthy volunteers and AD patients, enhanced hypertrophy of neuroglia and stellate cells called astrocytosis was demonstrated through the immunologic staining of glial fibrillary acid protein (GFAP) [57]. In aged laboratory animals, the age-related activation of glial cells manifested in an increase in the number of GFAP-positive astrocytes with a classic stellate morphology [39]. The activation of microglia in the cerebellum of aged mice, detected through the co-localization of the brain immunity modulator Pax6 with microglial cell-marker-ionized binding protein 1 (Iba1), suggests the intensification of inflammatory processes in the cerebellum [58]. In the cerebellum of aged mice, neuroglial cell activation may lead to alterations in lipid metabolism that are manifested by the enhanced expression of a lipolysis stimulated receptor (LSR) in glial cells. Enhanced LSR expression indicates the suppression of the synthesis and/or loading of cholesterol onto lipoproteins in the glia, resulting in abnormal protein and lipid trafficking, decreased synapse assembly in neurons, and consequent synaptic dysfunction [59]. Moreover, the cerebellum of aged mice showed enhanced spontaneous glial wave activity through the molecular and PC layers that are associated with low resting brain oxygen tension. These data might suggest the decrease in the interconnection between different cerebellar cell types during the aging process [60,61].

The mitochondrial AAA protease enzymes mediate the morphological and functional changes in cerebellar glial cells, secondary morphological degeneration, and electrophysiological changes in other cerebellar cells such as the PC [61]. Age-related total cerebellar volume loss is associated with a decrease in the number of PCs. The PCs of aged animals exhibit a noticeable decrease in the density, size, number, and length of dendritic branches, number of nodes, surface area, and process volume. These morphological changes in PCs aggravate chronic inflammation, as demonstrated in aged GFAP-interleukin (IL)-6 transgenic mice [39]. However, in other studies on the cerebellum of aged rat, the number of PCs and granule cells in cerebellar folia IV, VII, and X did not change significantly [62]. Instead, the volume of the molecular layer was reduced, and the number of dendritic spines and axonal and dendritic processes decreased [62,63]. In another study, aged rats demonstrated a loss of synaptic spine density in the cerebellar cortex. In the distal arborization of the PCs, the number of all dendritic spine morphologies (e.g., thin, mushroom, stubby, and wide spines) was proportionally lowered, which was accompanied by impaired motor and memory performance [64]. Interestingly, the age-related accumulation of lipofuscin causes an increase in PC volume [65,66,67,68,69]. In particular, in the cerebella of aged fat-tailed dwarf lemurs, lipofuscin accumulation was observed in the cell bodies of Bergmann glia, basket cells, and stellate cells, and PC dendrites in the ML [70]. In humans, aging leads to a loss of PC perikaryon volume, which might be linked to the limited deposition of lipofuscin in human PCs [71], relative to the abundant lipofuscin deposition in the PC of several mammalian species, as demonstrated by Gilissen et al. (2016) [72].

Significant decreases in the number of PCs during the aging process are mediated in part by apoptosis. In the cerebellum of aged rat, the number of p53-immunopositive neurons in the PC layer increases. In ischemia, the upregulation of the p53 gene is associated with hypoxia-induced apoptosis and lower neuronal viability. In the CNS, the PCs are among the cells that are the most vulnerable to hypoxia and ischemia, which may account for their apoptotic death during the aging process [73]. In mice with PC-specific knock-out of the DNA excision repair protein 1 (Ercc1), aging also upregulates the expression of several genes in the PC, including the glial cell marker connexin43, the astrocyte marker GFAP, the macrophage marker Mac-2, the negative apoptosis regulator metallothionein 1 (MT-1), the apoptosis mediator caspase-3, and the complement factor C1qC that aids in the removal of apoptotic cells [74]. Aged cerebellar granule cells exhibit an accumulation of the cell-death-associated endopeptidase prolyl oligopeptidase [75], which leads to increased inflammation and damage to cerebellar neurons. In turn, these might induce the intensification of oxidative stress-induced DNA damage [76]. No significant epigenetic changes such as histone H3 expression or occupancy were observed, as demonstrated in aged C57Bl6 mice [77]. Similarly, no significant acceleration of epigenetic aging was observed in the cerebellum of bipolar disorder patients, as measured using variations in genome-wide DNA methylation, mtDNA copy number, and telomere length [78]. This finding is in contrast to that observed in the PCs of aged mice exposed to moderate (15%) calorie restriction, where aging altered DNA methylation and hydroxymethylation, as demonstrated by a significant increase in their 5-mC and 5-hmC immunoreactivity. Remarkably, these age-related changes could be countered by severe (50%) calorie restriction [79]. In addition, the PCs of aged mice exhibit significantly lowered levels of the mRNA for the calcium-binding protein calbindin-D28k, indicating impaired functionality of the cerebellum [80]. Finally, the PCs of aged mice also exhibit the downregulation of proteins such as synaptic scaffold proteins (Shank2 and Homer-3), neurotransmitter receptors (GluRδ2, mGluR1, GABABR1, and GABABR2), signal transduction enzymes (cGK1, PKC-γ, IP3KA, CaMK-IIα, and Pde5a), and ion channels/transporters (TrpC3, SERCA3, Kvβ1, and Cavα2δ2), leading to synaptic dysfunction [74]. The downregulation of these proteins in an aged cerebellum is likely to result in gradual functional decline, tissue degeneration, and aberrant neurotransmitter signaling. 

In aged chickens (*Gallus gallus*), a significant reduction was observed in the mean density of PCs stained for the GluR1 and GluR1–3 subunits of the AMPA receptor that correlates with the diminished synaptic plasticity in the aged cerebellum and associated cognitive and motor impairments [81]. On the intracellular level, the PC mitochondria exhibited age-dependent decreases in volume, numeric density, and volume density [82,83]. Aged PCs also exhibit alterations of other organelles, including the dilation of the smooth endoplasmic reticulum [62], reduction in the nucleolar volume [84], changes in the nucleolar texture, repartition in the nucleolus, and redistribution of free ribosomes [85]. Such age-related intracellular changes in the PC might affect energy metabolism and intraneuronal homeostasis, resulting in apoptosis, which, in aged humans, leads to cerebellar dysfunction and motor deficits [86]. Aging is associated with frequent cytoplasmic mis-localization and a nuclear depletion of the RNA-binding protein heterogeneous ribonucleoprotein (hnRNP) K in the dentate nucleus of human cerebella [87]. Aged mice demonstrated a decrease in the density of NO synthase-expressing granular cells [88]. In the cerebellum of aged mice, the GCs, PCs, and molecular cell layer exhibited a reduced expression of Pax6 and Ras-GAP and a lower number of co-localized Pax6 and Ras-GAP cells. As Pax6 and Ras-GAP are crucial for the functioning of brain synapses, their loss might underlie a molecular mechanism for an age-dependent decrease in synaptic plasticity [89]. The cerebellar cortex of aging mice exhibited an intensification of DNA damage, as measured using the phosphorylation of histone H2AX, (γH2AX), which is temporally and functionally related to the proliferation and apoptosis of cerebellar granule cell precursors [90].

## 3. Role of the Cerebellum in Neurodegenerative Disorders

There is increasing evidence to suggest a role for the cerebellum in neurodegenerative disorders such as Alzheimer’s disease, Parkinson’s disease, Huntington’s disease, amyotrophic lateral sclerosis, Friedreich’s ataxia, spinal muscular atrophy, juvenile Batten’s disease, and multiple sclerosis, etc. What is known about the role of the cerebellum in these devastating disorders is discussed below.

### 3.1. Alzheimer’s Disease (AD)

The amyloid plaques characteristic of AD appear in the cerebellum primarily during the later stages of the disease [91,92]. The cerebellum is more resistant to the neurotoxicity caused by the soluble amyloid-beta (Aβ) protein, as shown by the lower levels of synapse loss in the cerebellum relative to other brain structures [93,94]. During the early stages of AD, the cerebellum compensates with enhanced activity that results in the improvement of some forms of memory [95,96] but the worsening of others, such as the topographic working memory that is functionally related to cerebellar memory [97,98]. The effects of AD on the cerebellum differ from those on other brain structures. For example, the GABAergic synapse and retrograde endocannabinoid signaling pathways are not downregulated in the cerebellum of AD patients [99], and in AD patients, the signs of neurological aging, including increased inflammation and reduced neuronal expression that are present in other brain structures, are absent in the cerebellum [100]. Interestingly, in familial AD, the earlier onset and greater severity of the disease correlate with cerebellar pathology [16]. In late-stage AD, noticeable cerebellar atrophy is demonstrated by an evident decrease in the volume of the ML and GC layers of the cerebellar cortex and a reduced number of PCs [101,102]. In the AD neocerebellum, the activation of microglia and development of neurovascular inflammation are not associated with Purkinje cell count or neuronal degeneration, as measured through immunoreactivity to tau and ubiquitin proteins [103]. Therefore, the presence or absence of neuronal degeneration in the cerebellum of AD patients is most likely associated with the degree of AD progression. This suggestion is supported by Pellegrini (2021), who showed that specific alterations of DNA methylation in the brain during AD are dependent on age, highlighting the accelerated epigenetic aging that occurs in AD [104]. This is consistent with the observation that pronounced changes in the memory performance of AD patients are associated with the extent of cerebellar damage, not the number of amyloid plaques [105]. The cognitive dysfunction in AD is likely to be related to accelerated cerebellar synaptic aging characterized by ultrastructural damage to synapses, including a loss of dendritic spines and synaptic boutons and a decrease in the number of synaptic vesicles and mitochondria in the presynaptic termini of cerebellar neurons [63]. Notably, a study performed in a mouse model of AD demonstrated electrophysiological alterations in the PCs and neurons of the DCN that were associated with a decrease in memory and poor performance in the water-maze test [106]. 

In addition to synaptic function, the efficiency of cognitive functions in the brain depends on the frequency and timing of neuronal spikes. Using the Purkinje cell model, it has been shown that the regulation of spike rate is mediated by rate-dependent subthreshold membrane potentials that trigger the activation of Na^+^ channels [107]. Cheron et al. showed that in mice with AD, there was evidence of electrophysiological changes in both PC and DCN, which could significantly contribute to behavioral deficits. These changes were associated with Aβ deposition in the molecular layer of the cerebellum [106]. 

### 3.2. Parkinson’s Disease (PD)

In PD, cerebellar dysfunction is associated with resting tremor, impaired balance, and unstable gait. PD is characterized by aggregations of alpha-synuclein protein in the pre-cerebellar brainstem and the cerebellar nuclei [108]. The severity of the resting tremor is associated with the iron content of the cerebellar dentate nuclei. Excessive cellular iron is a root cause of ferroptosis, a nonapoptotic cell death pathway that leads to the degradation of cerebellar neurons [109]. PD patients and patients with PD-PIGD (postural instability and gait disorders) exhibit cerebellar atrophy, specifically the reduced volume of the right Crus II, right lobules IV and V, lobules VIII and IX, vermis VIII and IX, pyramis, and culmen [110,111,112]. Changes in the volume of cerebellar lobules are typical for PD patients, where an increased volume of the lobule IV is positively correlated with resting tremor and the severity of total tremor, and a decreased volume of the lobule VIIb is positively correlated with a more severe tremor [113,114]. 

Functional MRI studies demonstrate the impact of cerebellar dysfunction on such PD symptoms as tremor and motor instability. Tremor-dominant PD patients exhibit reduced functional connectivity between the cerebellar dentate nucleus and the ventral lateral posterior nucleus of the thalamus [115], the sensorimotor cortex and vermis [116], the dorsal attention network, and the cerebellar somatomotor network [117]. However, tremor-dominant PD patients converting to the more aggressive PD-PIGD form demonstrate increased functional connectivity in the temporal and occipital lobes and in the cerebellum and pontomedullary junction relative to patients who do not convert to PD-PIGD [118]. PD-PIGD patients also demonstrate increased activity in non-motor cerebellar areas during gait-simulating tasks, most likely as a compensatory response to the functional failure of the motor areas of the cerebellum and basal ganglia [112]. PD patients who experience gait freezing during lower-limb movements demonstrate reduced theta oscillations and attenuated cue-triggered theta-band power via the mid-cerebellar Cbz electrode in electroencephalography (EEG) studies during motor tasks [119,120]. 

One of the most important complications of PD is cognitive decline, a hallmark of poor prognosis. In non-amnestic PD patients, cognitive function studies using MRI studies have shown that cerebellar lobule VII participates in networks within the visuospatial-executive and attention domains and have suggested that lobule VII may play an important role in the development of PD cognitive decline [121]. PET studies on the cerebellum in PD patients demonstrate increased local glucose metabolism in the posterior cerebellar vermis that is associated with impaired memory, attention, and executive function [122]. Similarly, increased glucose metabolism in the right crus I, crus II, and vermian lobule VI is associated with the severity of cognitive impairment [123].

### 3.3. Huntington’s Disease (HD)

HD is characterized by uncontrollable movement (chorea), abnormal body posture, and functional deficits in behavior, cognition, emotion, and personality changes. In HD, pronounced cerebellar degeneration was observed in the right Crus I lobule, bilateral Crus II lobules, and the left VIIb and VIIIa lobules [124], while another study noted significant volume loss in the cerebellar grey and white matter [125]. Cerebellar volume loss is associated with a reduced number of PCs and GCs [126,127]. Notably, there was a detrimental change in gene expression, particularly of those factors that are responsible for exocytosis and vesicular fusion in GCs [128].

### 3.4. Amyotrophic Lateral Sclerosis (ALS)

ALS, also called Lou Gehrig’s disease, is a fatal motor neuron disease associated with progressive cerebellar degeneration [129]. The specific role of the cerebellum in this disorder may be related to the cerebellum-specific repeat expansion of several genes that determine ALS pathology, including the NIPA magnesium transporter 1 (*NIPA1*), chromosome 9 open reading frame 72 (*C9ORF72*), and ataxin-1 (*ATXN1*) genes [130]. The localization of ALS-related cerebellar atrophy varies. For example, sporadic ALS patients demonstrate atrophy of lobules I-V in the cerebellar anterior lobe. At the same time, carriers of the *C9ORF72* gene mutation (expansion of a GGGGCC repeat to as many as 1600 copies) demonstrate atrophy of the posterior lobe and vermis, while patients with intermediate expansions in the *ATXN2* gene do not demonstrate significant cerebellar atrophy [131].

### 3.5. Friedreich’s Ataxia (FRDA)

FRDA is a hereditary disorder characterized by progressive motor dysfunction, altered tactile sensation/sensitivity, and impaired speech [132]. The cerebellum of FRDA patients exhibits a moderate reduction in volume and patchy atrophy, especially of the dentate nucleus and Lobule IX, based on the results of MRI studies [133,134]. Mild atrophy of the medial parts of lobule VI may be related to speech impairment [133]. Moreover, an impaired visuospatial function in FRDA is directly correlated to the volume of cerebellar lobule IX [135]. Voxel-wise seed-based functional connectivity fMRI analysis was used to show that connectivity between the anterior cerebellum and bilateral pre/postcentral gyri and between the superior posterior cerebellum and left dorsolateral prefrontal cortex (PFC) was significantly reduced and that these changes correlated with disease severity [135]. Changes in cerebellar morphology in FRDA patients may result from chronic inflammation and glial activation in the dentate nuclei [132]. In a mouse model of FRDA, the affected animals exhibited loss of cerebellar PCs and principal neurons of the large dentate nuclei and selective degeneration of climbing fiber synapses, identified using the glutamatergic synaptic marker VGLUT2 [136].

### 3.6. Spinal Muscular Atrophy (SMA)

SMA affects spinal cord motor neurons, causing their atrophy from inactivity. The development of the loss of motor control in SMA patients involves the neurodegeneration of several brain regions, including the cerebellum [137,138]. SMA patients exhibit volume loss of cerebellar lobules VIIIb, IX, and X and gray matter atrophy of lobule IX, as determined through MRI studies without correlation with clinical manifestation [139]. In a mouse model of SMA, affected mice demonstrated neuronal dysfunction of the cerebellum that was manifested in lower spontaneous firing and lower cerebellar network activity, most noticeably, lower spontaneous excitatory and inhibitory synaptic activities of cerebellar PCs, relative to the control animals [140].

### 3.7. Juvenile Batten’s Disease (JBD)

The progressive neurodegenerative disorder JBD, also known as the juvenile form of neuronal ceroid lipofuscinosis (JNCL), is characterized by defects in lysosomal storage caused by mutations in the ceroid lipofuscinosis neuronal 3 (*Cln3*) gene, leading to the degeneration of cerebellar and retinal neurons [141]. Cerebellar atrophy in JBD causes motor function deficiency, disturbed balance and coordination, and abnormal EEG findings [142,143,144,145]. The severity of cerebellar atrophy positively correlates with the level of dysfunction [143]. Although earlier studies on the *Cln3*-knockout mouse model for JBD showed that the dysregulation of the granular cell AMPA receptors played a role in cerebellar degeneration [144], while more recent studies demonstrated presynaptic changes in the mossy fibers that project from BS/SC to the cerebellar IGL (Figure 1) but did not observe postsynaptic AMPAR dysfunction [141].

### 3.8. Multiple Sclerosis (MS)

In patients with MS, cerebellar dysfunction starts in the early stages of the disease [146]. The walk ratio (WR) of step length/cadence serves as a speed-independent index of the neuromotor control of gait [147]. During the development of ataxia, the volume of the cerebellum correlates with the walk ratio [148]. An analysis of the cerebellar damage in MS patients using MRI showed that the reduced volume of cerebellar lobules I-IV, reduced volume of GM in the lower vermis, lesions of the cerebellar superior peduncle, and increased volume of GM in cerebellar lobules VIIIb and Crus II correlate with physical disability and cognitive disfunction [149]. Another MRI study of MS patients showed a relationship between cognitive deficiency and diminished cerebellar functional connectivity, where patients with secondary progressive multiple sclerosis (SPMS) showed the most severe cognitive impairment and cerebellar damage and exhibited changes in cerebellar functional connectivity [150]. A separate MRI study showed an association between reduced functional connectivity in the cerebellum, greater loss of cerebellar volume, and more severe lesion burden. And a loss of white matter volume reduced functional connectivity in the sensorimotor cerebellum lobes, while a loss of cortical grey matter volume was linked to increased connectivity of both sensorimotor and cognitive cerebellum tissue [146]. 

An assessment of postural sway deficits and cerebellar peduncle lesions in MS patients using diffusor–tensor imaging (DTI) showed that peduncles in the inferior cerebellum contribute to the control of standing balance with visual input (somatosensory information), while those in the superior cerebellum contribute to reactive balance control without visual input. Notably, MS patients exhibit cerebellar lesions even at the prodromal stage of the disease, when cerebellar motor symptoms have not yet appeared [151]. Interestingly, patients who participated in a 12-week high-intensity balance training program exhibited improved balance due to changes in postural sway and DTI parameters. The mechanism by which such improvement occurs is believed to be compensatory training-induced transient structural plasticity in the white matter (WM) tracts that form the cerebellar peduncles, resulting from improved myelination. Although the changes in clinical findings and imaging parameters persisted only for the 12 weeks of the training program, these results provide hope that high-intensity task-oriented exercises will prompt positive changes in the cerebellar microstructure and improve the clinical outcomes of MS [152].

### 3.9. Vascular Dementia (VD)

Cerebrovascular diseases, especially VD, are increasingly prevalent in aging, tremendously impacting a patient’s quality of life. Cerebrovascular diseases such as stroke can impact the cerebellum due to cerebellar bi-directional interconnection to numerous brain regions, severely decreasing cerebellar function. Conversely, the activation and consequent increase in the function of the cerebellum can effectively alleviate symptoms of a disease such as vascular dementia [153]. Nevertheless, the precise mechanism by which metabolic and circulatory disturbances in the cerebellum affect cognition remains to be uncovered. 

Evidence suggests that individuals with cerebral small vessel diseases may develop compensatory cerebellar hyperconnectivity in the regions that relate to the frontoparietal cognitive networks and sensorimotor areas of the brain, demonstrating hypoconnectivity in frontoparietal brain regions [154]. On the contrary, Ruan and colleagues demonstrated that individuals with vascular mild cognitive impairment in certain areas of the cerebellum demonstrated significantly decreased functional connectivity between the cerebellum and regions of the brain in the default mode network, sensory-motor network, and frontoparietal network [155]. It has been demonstrated that subcortical vascular mild cognitive impairment is associated with anatomical atrophy and reduced functional connectivity to the striatum in specific cerebellar regions that are involved in cognitive function [156].

Quantitative MRI in VD indicates generalized cerebellar atrophy [157]. Additionally, various studies have proposed that cerebellar-mediated cognitive decline is more severe in VD patients than in those with AD [158]. It is speculated that greater cerebellar atrophy in VD may serve as a valuable diagnostic marker to differentiate between VD and AD [159,160].

Morphological alterations in the cerebellum during VD result from the increased susceptibility of Purkinje cells to ischemia. While the precise mechanisms of cerebellar atrophy in hypoxia are not clear, there is evidence that VD leads to a reduction in cerebellar metabolism [161], which is not a typical trait among AD patients [162]. Mielke’s research showed that the regional cerebral glucose metabolism rate in the cerebellum dropped solely in VD patients but not in those with AD [163]. Postmortem studies by De Reuck et al. support the specific role of VD in cerebellar damage. Brain samples from patients with AD, AD associated with cerebellar amyloid angiopathy, frontal degeneration, amyotrophic lateral sclerosis, Levi’s disease, progressive supranuclear palsy, and VD were studied. The findings indicate an appreciable rise in microhemorrhages and microinfarcts in the cerebellum solely among VD patients [164].

Experimental studies confirmed the role of hypoxia in cerebellar damage. A mouse model of VD demonstrated increased expression of inflammasome receptors, adaptor, and effector proteins, markers of inflammasome activation, proinflammatory cytokines, and apoptotic and pyroptotic cell death proteins in the cerebellum [165].

Ischemic white matter damage is considered a significant factor in cognitive decline [166]. Cultured organotypic slices of the cerebellum were utilized to create an in vitro model of chronic ischemic white matter damage. Prolonged hypoxic injury was employed to accurately reproduce the predominant axonal degeneration. This model enables scientists to elucidate the mechanisms of cerebellar atrophy in hypoxia and to effectively test potential drugs that can attenuate axonal degeneration.

## 4. Conclusions

The cerebellum, which mediates movement, cognition, and learning, is tremendously important for normal, day-to-day human activities throughout the lifespan. The proper development of the cerebellum provides the basis for its future healthy aging, while impaired cerebellar function during the aging process or as a result of neurodegenerative disorders leads to devastating clinical manifestations that decrease the quality of life (QOL). Uncovering the mechanisms of cerebellar aging and the development of neurodegenerative diseases that affect the cerebellum is of utmost importance for developing novel therapeutic approaches to improve the overall human QOL.
